# The perception of prosocial agents by chimpanzees and humans

**DOI:** 10.1098/rsos.250916

**Published:** 2025-11-12

**Authors:** Sarah Brocard, Chloé Berton, Vanessa Wilson, Balthasar Bickel, Klaus Zuberbühler

**Affiliations:** ^1^ Department of Comparative Cognition, University of Neuchâtel Faculty of Sciences, Neuchatel, Switzerland; ^2^ School of Psychology and Social Work, University of Hull, Hull, UK; ^3^ Institute for the Interdisciplinary Study of Language Evolution (ISLE), University of Zurich, Zurich, Switzerland; ^4^ School of Psychology and Neuroscience, University of St Andrews, St Andrews, UK

**Keywords:** prosociality, affective perception, social perception, agent preference, event cognition

## Abstract

The human propensity for prosocial behaviour has no equal, not even in our closest living relatives, the chimpanzees. However, it remains unclear whether this difference is grounded in the sheer perception and cognitive evaluation of prosociality. We investigated how two hominid species, chimpanzees and humans, perceive third-party social interactions with prosocial, neutral and antisocial agents. Using a touchscreen paradigm, human and chimpanzee participants freely selected between two actors after viewing their interactions, ranging from pro- to antisocial. Contrary to current thinking, we found no evidence for species differences in their choices for agents, regardless of whether interactions were between conspecifics or not. Both humans and chimpanzees demonstrated comparable sensitivity to prosociality, challenging existing views of a profound chimpanzee-human difference in prosociality. Instead, our results indicate that the perception of social interactions is similar across hominids, but that humans have evolutionarily diverged in how they act upon such perceptions.

## 1. Introduction

Social interactions can broadly be categorized based on their impact on the recipient, ranging from antisocial (e.g. aggressive, deceptive), to neutral (e.g. co-feeding), to prosocial (e.g. affiliative, helpful). As an apparent Darwinian paradox, prosocial behaviours are of special interest for evolutionary theory and are loosely defined as ‘...behaviours that are intended to benefit others’ [p.748; 1]. A large spectrum of behaviours can fall into this category, depending on context, provided there is some sort of benefit to the recipient. To address this, many researchers therefore specifically require prosocial behaviour to be (seemingly) voluntarily and intentionally performed, rather than accidentally deployed [2], regardless of goals, gains or costs [2,3].

Prosocial behaviour may also be seen during cooperative behaviour where two or more individuals work together towards a common goal. For example, social carnivores rely on each other to hunt or defend resources [4,5], cleaner fish cooperate with their clients [6,7], meerkats cooperate to defend their territories [8] or common marmosets cooperate to raise their offspring ([9,10]; see [11] for a review). However, humans go far beyond such task-specific cooperative interactions with immediate mutual benefits and often help others without any obvious gain, even for the foreseeable future. In addition, they effortlessly and routinely engage in joint activities and cooperate easily with unrelated and even unknown individuals, often prioritizing joint efficiency, which allows them to cooperate at large scale, something referred as hyper-cooperation [12–17].

How did human hyper-cooperation evolve? One prominent proposal is that this was enabled by an evolutionary change in human psychology to include a basic prosocial motivation [14]. From early on, human infants recognize and act upon the emotions of others [18,19] and show high commitment and motivation to engage in cooperative interactions and expect reciprocity in return [20–23], even without immediate benefits [14,24–27]. Human prosocial behaviour, in other words, appears to be an emancipated form of cooperative behaviour, executed unilaterally and without the need of immediate reciprocation. The underlying driving force, prosocial motivation, can stem from empathy, moral principles or social norms [28]. Regarding empathy, chimpanzees appear to possess some key ingredients as they can exhibit attentional biases towards emotionally charged stimuli [29–32], remember and recognize aggressive expressions over neutral ones [33] and distinguish facial expressions [34] and other forms of affective expressions [35–37].

These findings have led to the more general question of whether prosociality is uniquely human or whether precursor abilities are present in other hominids. Wild chimpanzees help each other in a number of ways, although most observations suggest some mutual benefit, such as dyadic or polyadic grooming [38,39], territory defence [40], coalitionary attacks [40], group hunting [40–42] or adopting orphaned group members [43,44]. In some instances, however, the observed behaviour appears to unilaterally benefit another, such as when informing other group members of deadly threats [45,46].

In captivity, the evidence for prosociality is less clear and often contradictory. Captive chimpanzees sometimes help or console each other [47–50] or provide resources at an individual cost [51]. They pick partners based on previously observed social behaviour [52], reward help [53–56], settle conflicts between others [57] or respond faster in cooperative rather than selfish tasks [58]. However, there is also evidence that chimpanzees sometimes remain spectacularly ignorant about others’ needs [59–61] and differ from humans in key aspects of cooperative behaviour [62,63]. For example, Duguid *et al*. [25] found that children increased their communication efforts if a cooperative task risked failure, whereas chimpanzees simply lost interest. Hence, although chimpanzees do cooperate, they appear to do so mainly to maximize their own personal benefits and, if this is not possible, prefer to act on their own [64], often irrespective of others’ needs [59–61]. Also striking are results from bonobos (*Pan paniscus*), who—when given a choice between a helping and a hindering agent—favoured the hindering, antisocial agent [65].

The question of prosociality and reciprocity in non-human primates, in other words, is still unsettled [66] and requires further research. One possible source for the difference between wild and captive chimpanzees is that, in captivity, individuals do not depend much on each other for survival, which might predict little prosocial behaviour (e.g. interdependence hypothesis [67]). However, the same argument could be made for humans, who nevertheless show prosocial behaviour even towards complete strangers, suggesting a fundamental species difference in prosocial motivation [22,68] and different evolutionary history [14,67,69].

In this study, we focus on another potential source of human-animal difference, i.e. how prosociality has been operationalized. The experimental literature that serves as the foundation for current theories mainly consists of action-based paradigms that request subjects to respond behaviourally to cooperation problems presented to them, mostly to get food rewards. Experiments can broadly be divided into assistance-providing tests, whereby a subject decides whether to assist another who needs help [27,70–72] or benefit-choice tests, whereby a subject chooses between actions that differ in their beneficial effects on another [59–61,73,74]. Results differ with assistance-providing tests generally providing more prosocial responses than benefit-choice tests. Yet, decisions made about who or when to help do not necessarily reflect the ability to perceive what is prosocial. Chimpanzees may share with humans the ability to recognize prosocial behaviours but simply act on this information differently. Given how important perception of social information is in decision-making [75,76], the key to understanding the species differences in social cognition may lie in how prosocial behaviours are perceived.

The present study investigates how chimpanzees and humans perceive prosocial interactions. Using natural agent/patient interactions (doer versus receiver), we let subjects freely choose one of two actors on a touchscreen device [77], following a brief video clip of a social interaction, previously rated in degrees of prosociality. Choices were interpreted as inherent attentional preferences for one actor over another, after adjusting for natural variations within the events (e.g. size of actors, motions).

If humans were profoundly different in terms of their prosocial psychology, then we predicted a stronger preference for prosocial agents compared to chimpanzees and the reverse pattern for antisocial agents [20,65]. Additionally, we anticipated that chimpanzees would favour antisocial over neutral agents, given their attentional bias for agonistic interactions and aggressive faces [29,30,32]. Besides, if prosociality perception was species-specific, we expected these effects to be more pronounced in interactions involving conspecifics.

## 2. Methods

### 2.1. Data collection

#### 2.1.1. Subjects

##### 2.1.1.1. Humans

Sixteen undergraduate students (*n* = 13 females; mean 23.1 ± 2.66 years old; range 18–28 years); from the University of Neuchatel (Switzerland) were recruited via email. The gender imbalance of the sample reflects that of the chimpanzee group and is unlikely to have biased the results, as a previous study using a similar paradigm found no sex difference in responses [77]. Before the start of the experiment, participants were informed that they would be watching short video clips depicting one individual interacting with another. They were then instructed to touch one individual on the screen once the video ended. Importantly, we stressed that there was no right or wrong answer. We provided this information to reduce the human tendency of overthinking and generating theories and predictions about the study’s purpose. This way, we assumed, we were more likely to capture the intuitive preferences of human (and chimpanzee) subjects regarding the different social situations presented to them. All human participants were naive to the purpose of the study, signed an informed consent form, completed a short questionnaire followed by a debriefing and ‘question and answer’ session after the experiment. Participants were then rewarded with a voucher worth CHF 22.00.

An additional 30 new participants affiliated with the University of Neuchatel (*n* = 20 females) were recruited via email to rate the prosocial nature of the video clips. These participants were informed of the purpose of the main study before completing a questionnaire and rating the social interactions of the videos.

##### 2.1.1.2. Chimpanzees

We were able to test seven voluntarily participating chimpanzees (*n* = 5 females; mean 15.14 ± 15.03 years old; range 3–42 years) housed at Basel Zoo (Switzerland), who were part of a cohesive social group consisting of 15 individuals at the time of the study (June 2021 to April 2022: one adult male, six adult females, one juvenile female, two infant males and five infant females). From the seven chimpanzees that started the training, four (*n* = 2 females) reached the preset criteria to be included in the test; the remaining three individuals (two adults and one juvenile) either lost interest (*n* = 1) or never reached criteria (*n* = 2). The remaining members of the group never showed interest in participating in this or any of our previous experiments (see [77–79]). All individuals had access to two outdoor enclosures (totalling 477 m^2^) and five indoor enclosures (totalling 290 m^2^) with a roof made of sliding glass windows ensuring natural lighting throughout the day. All enclosures were provided with ropes, hammocks and climbing structures, and fresh daily material to build nests. Individuals were fed a mix of fruit and vegetables, supplemented with small amounts of protein, with several feeds distributed throughout the day and constant free access to water.

### 2.1.2. Procedure

Training and testing data were collected every weekday from 8.00 to 18.00 for the human participants and from 8.00 to 11.00 for the chimpanzees. We used the same touchscreen devices for all subjects (Iiyama ProLite T1931SR, 19", 48 cm and 1280 × 1024 resolution, using resistive technology) connected to a laptop (Dell Latitude 7420). The touchscreens were calibrated every morning before the start of the experiment with eGalaxTouch software (v.5.14.0.19810). The experiment was programmed and displayed using Matlab R2017a (v.9.13.0) software and specifically Psychophysics Toolbox v. 3 (PTB-3) extensions [80,81]. The chimpanzees’ set-up consisted of the touchscreen encased in a Plexiglas box (73 × 55 × 35 cm) fixed in one of the indoor enclosures. The plexiglass box had a large opening (16.5 × 32.5 cm) at the bottom that allowed the chimpanzees to touch the screen and retrieve rewards. The set-up was always accessible to all individuals but was only operational during testing sessions when the experimenter was present.

**Figure 1.**
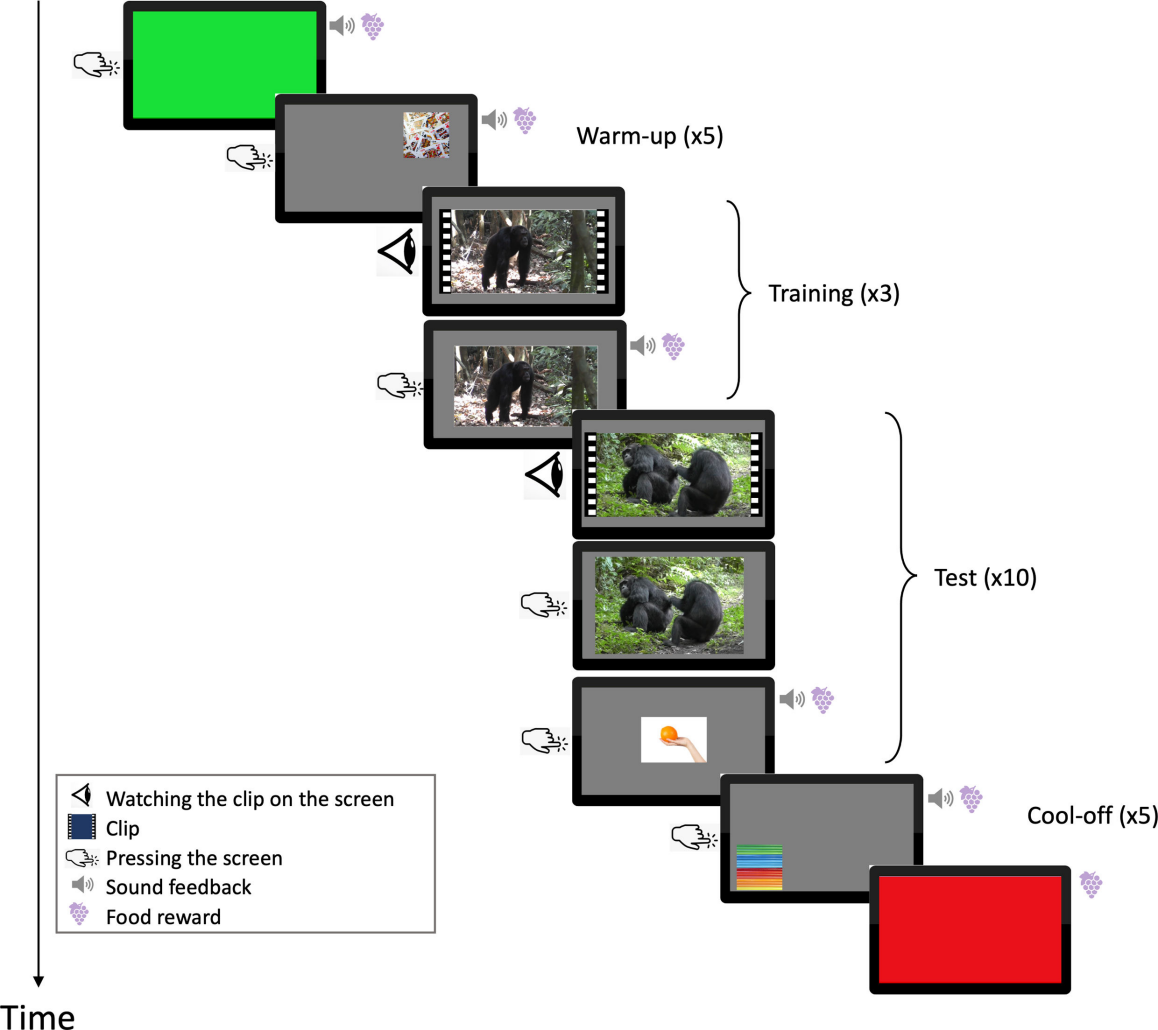
General procedure of each session for chimpanzees, starting with a green screen and ending with a red one. Following the green screen, subjects went through *n* = 5 warm-up trials (random images of 380 × 380 pixels) and *n* = 3 training trials (electronic supplementary material, figure S1), followed by (usually) *n* = 10 test trials. Subjects watched the clip to the last frame, presented to them as a still image. They then chose one of the two actors after which the still image disappeared for another image, a human hand holding a fruit (600 × 600 pixels). After the test trials, the subjects went through *n* = 5 cool-off trials (random images of 380 × 380 pixels) and the final red screen. The clips were played in full screen mode (1280 × 1024 pixels). Positive feedback sounds and food rewards were given after subjects correctly pressed within images. If subjects touched the screen outside the image, a negative feedback sound was emitted, and they had to try again until they succeeded. The human participants also started with a green screen but then directly moved to the tests and finished with the red screen after watching all test trials. Instead of a hand holding the fruit, human subjects saw images of an arrow after they pressed on the video’s still image. Photo credits: A. Soldati and C. Hobaiter.

Chimpanzees were never isolated from the social group, and daily testing sessions started when an individual voluntarily approached and pressed a green screen, which was followed by a food reward. We then continued with five warm-up trials (images randomly positioned on the screen) and three training trials (see the electronic supplementary material, S1 and figure S1). Then, participants saw approximately 10 experimental trials and finally five cool-off trials and a uniformly red screen at which point the participant obtained three pieces of grape (figure 1). For humans, sessions also started with a uniformly green screen, immediately followed by the test trials and a uniformly red screen at the end. Here, exposure was not limited to 10 trials, but all clips were shown in a row, with up to two 5 min breaks in between if requested.

To avoid inducing bias in the participants’ choices, each test trial was decomposed into three parts: participants (i) watched the clip, (ii) selected one actor on the last frame, and (iii) pressed on an image (a human hand holding a fruit for the chimpanzees and an arrow for the humans) to get a positive sound, as well as a food reward for the chimpanzees (figure 1).

**Figure 2.**
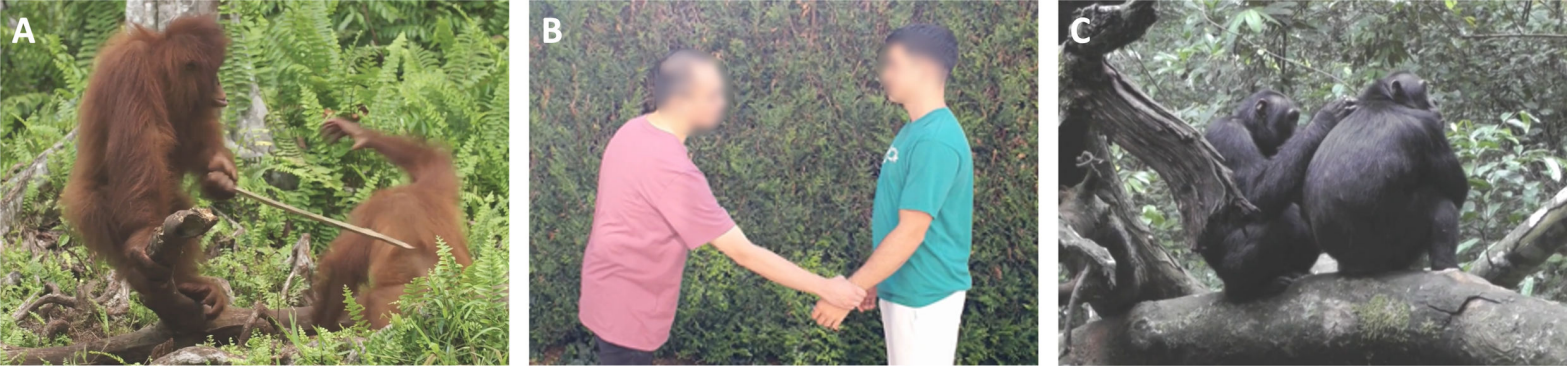
Stimuli examples rated by humans as (A) antisocial, (B) neutral and (C) prosocial events. In all these images, the agent stands on the left-hand side of the image. In (A) the agent hits the patient with a stick; in (B) the agent gently grabs and lifts the arm of the patient before releasing it, and in (C) the agent grooms the patient. Photo credits: (A) J. Curran, filming crew of Orangutan Jungle School Season no. 1 and Love Nature, (B) C. Berton, and (C) A. Soldati and C. Hobaiter.

Test trials consisted of short video clips displaying two individuals who interacted with each other as part of a natural social event (*n* = 200). All video clips were approximately 5 s long (electronic supplementary material, table S1), soundless and coloured. The social interaction events encompassed a range from prosocial to antisocial behaviours, including aggression, touching, grooming or playing (figure 2; electronic supplementary material, table S2). We also added a substantial number of control events (*n* = 75), where actors did not interact. The two actors featured in a clip were always from the same species but could differ in other features (e.g. size, sex, position on screen, motion). Given that previous research has shown both humans and chimpanzees are sensitive to expressions of inner states of both conspecifics and heterospecifics and similarly attend to their social interaction [29,30,36,78,82,83], we opted for showing video clips of different species. To this end, clips were sorted into five blocks (15 controls and 40 tests) according to the species of the actors: humans, chimpanzees, gorillas, orangutans and non-primates (see the electronic supplementary material, table S1 and also for details and examples of the clips). We included non-primates to test whether eventual prosocial preferences generalized to any animated agent or whether they were limited to hominids. In total, each subject went through 275 trials (200 tests and 75 controls). Each testing clip was presented in its original version or in its mirror one, to counterbalance the side of the agent across trials.

### 2.1.3. Regions of interest

To define the regions of interest (ROIs), the last frame of the video was extracted (from the original version of the video, not the mirrored one) and the abstract silhouettes of the actors were manually drawn. In case of contact between the two actors, the ‘action’ area was excluded (approximately 50% of the stimuli), as we wanted to test choices for either agent or patient. Additionally, if one actor was considerably smaller than the other, the size of the smaller actor was artificially increased within its ROI (something that was controlled for in the analyses), ensuring fairness for both actors. The ROIs of experimental stimuli were then flipped to generate the mirrored versions. The ROIs were not visible on the screen but were used by the programme to identify choices made by the participants.

### 2.1.4. Rating of prosociality in the stimuli

The survey was designed with Google Form and was divided into three parts. First, the 30 additional participants were introduced to the purpose of the study and were given definitions and policy. Then, participants were asked to fill out a questionnaire. The main goal of this was to assess the degree of participants’ knowledge of animal behaviour, which may impact their ability to determine a behaviour as prosocial in other species. Finally, participants had to rate 175 of the 200 video clips used in the main experiment. Very similar videos of the same species (e.g. two chimpanzees grooming in a green patch of the wood) were removed. The videos were judged with a 6-point Likert scale (where 1 indicated totally disagree and 6 indicated totally agree), based on the assertion: ‘The video showed an individual that performed a positive action which provided benefits (self-care, food...) to the other individual’. This 6-point Likert scale was chosen to prevent participants from neutral choice and to provide them with an ample range of options to respond in the most impartial manner possible. It took approximately 40 min to complete the entire survey, but participants were free to end and resume as it suited them.

### 2.2. Analysis

Statistical analyses were conducted using R, v.4.0.5 [84].

#### 2.2.1. Interrater reliabilities of videos

The interrater reliabilities of videos were examined using intraclass correlation coefficients (ICCs [85]) and their 95% confidence interval. The reliability of mean ratings across *k* raters was estimated using ICC(3,*k*), which corresponds to a two-way mixed-effects model of the consistency of multiple raters (30 in the current study) per measurement [86]. According to Koo & Li [86], results revealed excellent reliability (ICC(3,*k*) = 0.980, 95% CI = [0.979; 0.982]).

#### 2.2.2. Model fitting

We specified Bayesian regression models with the help of the *brms* package [87–89] interface to Stan [90]. We chose a Bayesian approach primarily because it outperforms traditional frequentist approaches in handling small sample sizes [91], as it accounts for all sources of measurement uncertainty. Specifically, Bayesian methods require the specification of prior beliefs about the data, which can help mitigate the potential impact of limited data by preventing estimates from being excessively influenced by small amounts of information. Furthermore, Bayesian models can not only estimate the probability of an effect but also the probability of no effect. This mitigates against the bias for reporting differences between species and under-reporting similarities, an issue that has hampered progress in the field [91].

To assess the effect of (i) the species, (ii) the mean prosociality rating of the video clips, and (iii) the species of the actors on the choice for the agent of the event, or the side of the choice during control events (see the electronic supplementary material, S2), we fitted Bayesian multi-level Bernoulli models with logit links.

In the full model (equation 2.1), these three focal parameters (the participant’s species, the mean rating of the clips and the species of the actor treated either as conspecifics/heterospecifics or as their actual species) were included as fixed (‘population-level’) effects. To adjust for potential confounding factors, the different features of the clip (e.g. the side of the agent, the difference of movement between the actors, the relative size of the actors in terms of rating the biggest actor or the actor that was the most centred on the screen on the last still frame; see the electronic supplementary material, table S3 for details and coding of these features) were included as additional fixed effects. Participant identity was included as a random (‘group-level’, varying) effect with varying slopes for the different clip features predictors and the clip identity was furthermore included as a random intercept (equation 2.1). Having the predictors relating to the clips’ features as both fixed and random effects helped with model fitting even if their effects were not of main interest. Formal definition of the full model:


agentchoice∼Bernoulli(p)logit(p)=(β1+ɑ[ID,1])×species+(β2+ɑ[ID,2])×conspecifics+(β3+ɑ[ID,3])×meanrating+β4×(species×conspecifics×mean rating)+(β5+ɑ[ID,4])×side choice+(β6+ɑ[ID,5])×side agent+(β7+ɑ[ID,6])(2.1)×trial+(β8+ɑ[ID,7])×status event+(β9+ɑ[ID,8])×agentbigger+(β10+ɑ[ID,9])×agentcentred+(β11+ɑ[ID,10])×agentmovelast+(β12+ɑ[ID,11])×differencemovement+γ[clipname,1]β indicates a fixed effect, while α and γ indicate random effects. The numerical subscripts link to each predictor.The term ɑ[ID, i]means that each level of each effect can vary by each participant’s ID, while γ[clip name,1]represents the random intercept by video clip. The part in blue corresponds to the focal parameters that are removed in the null model and that vary across models we compare (i.e., with or without interaction, with or without these predictors and with or without splines for mean rating). 

Weakly informative Normal (*μ* = 0, *σ* = 1.5) priors were used for all fixed effects, while exponential (*λ* = 1) priors were used for the standard deviation of random effects, and all models were fitted without intercepts. To test the sensitivity of inferences to prior choices, the best fitting model was refitted using priors with wider variance *N*(*μ* = 0, *σ* = 10) and narrower variance, *N*(*μ* = 0, *σ* = 0.5). The results remained consistent, suggesting that our results are not artefacts of the chosen priors (electronic supplementary material, figure S2).

We assessed the overall relevance of the three focal parameters by comparing the predictive performance of models with or without them and with or without interactions between them. Importantly, across all models, we only varied the focal parameters. In addition, we explored the possibility that participants might express a preference for both prosocial and antisocial agents, but no preference for more neutral agents, a pattern that would result in a U-shaped relationship. To model these potential nonlinear effects, we used thin-plate splines, as they flexibly capture complex patterns by minimizing function curvature and offer greater adaptability to the data while avoiding overfitting [92].

We assessed predictive performance of models in terms of the expected log-pointwise predictive (elpd) in leave-one-out cross-validation, i.e. the ability for a model to accurately predict unseen data points [91,93]. For efficiency, we approximated elpd estimates by importance sampling from the posterior [93]. When the difference in elpd (Δelpd) was ≤ 0.5 and the standard error of the difference (s.e._Δelpd_) exceeded Δelpd, we considered models to fit the data equally well, in which case we preferred the more parsimonious model, with fewer parameters.

To further quantify the weight of parameters in predictive performance, we additionally used the model stacking method [94], an alternative to information criteria such as Akaike information criteria and its variants [95,96]. Model stacking assigns weights to individual models to optimize joint predictive performance, where each model’s weight reflects its relative contribution to maximize the log-likelihood in cross-validation.

Finally, we reported the posterior estimates for each focal parameter (means, standard deviation and the 90% credible interval). We visualized these estimates with plots of the entire posterior distribution along with the 90%, 60% and 30% credible intervals. A preference in agent choice was declared as ‘robust’ if at least 90% of the posterior estimates were above a random or absent choice, i.e. above a 0.5 probability (or 0 on a logit scale) of agent choice. Average marginal effects (i.e. the average change in the predicted outcome when a parameter changes, averaging over the distribution of the other covariates) were also computed with the *marginaleffects* package [97].

Convergence of all models was controlled with trace plots, R-hat values and effective sample size diagnostics. Both prior and posterior predictive checks were further used to visually compare the observed data with the data simulated by the model. Overly influential data points were identified through Pareto *k* estimates during cross-validation with importance sampling [93,94]. These values indicate the level of bias introduced by the approximation and help identify influential observations in the model. All Pareto *k* estimates were within the commonly accepted range (*k* < 0.7), suggesting that there were no overly influential data points and that our elpd estimates were reliable.

## 3. Results

### 3.1. Preference for prosocial agents

A comparison of all models revealed that the null model was consistently outperformed by models including the predictors of interest (stacking weight = 0; Δelpd = −10.2, s.e._Δelpd_ = 4.0, null model relative to the best fitting model; see the electronic supplementary material, S4 for details). This comparison also revealed that adding complexity by modelling nonlinear relationships (i.e. splines) did not substantially improve model fits (see the electronic supplementary material, table S4, figures S3 and S4), indicating that the effect of prosociality was linear.

Among the models with the predictors of interest, several fitted the data equally well (see the electronic supplementary material, table S4 and figure S4). However, following a parsimonious approach, the model including only the mean prosociality rating of the event was considered the best (Δelpd = −0.4, s.e._Δelpd_ = 0.9, relative to the model with the lowest elpd; stacking weight = 0.108), suggesting that the species of the participants and conspecificity of the actors had only minor influence on the choices compared to the perceived prosociality of the events. Analysis of this second-best model revealed that the likelihood of selecting the agent slightly and linearly increased with the (human-rated) prosociality of the interaction (average increase of agent choice probability by mean rating: mean $\frac{\partial P\left(agent\;choice\;|\;mean\;rating\right)}{\partial \left(mean\;rating\right)}$ = 0.027, 90% CI = [0.013; 0.041], P$\left(\frac{\partial P\left(agent\;choice\;|\;mean\;rating\right)}{\partial \left(mean\;rating\right)}>0\right)$ = 1).

**Figure 3.**
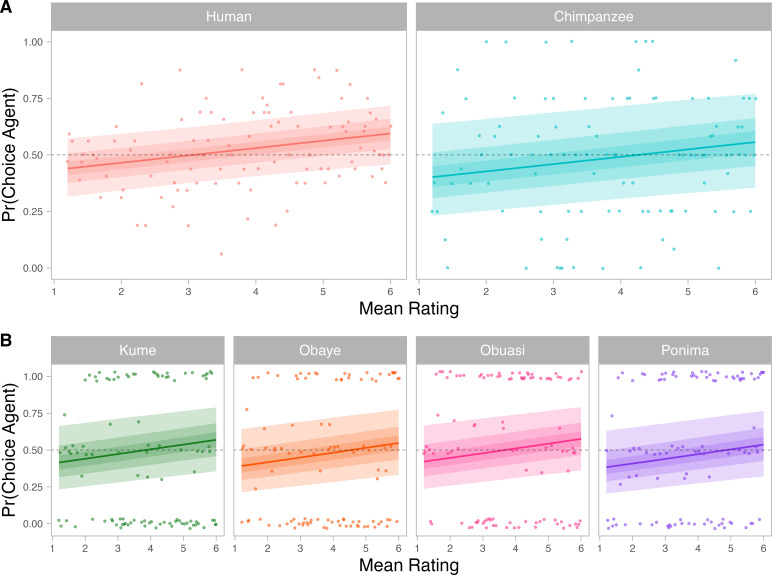
Posterior probability of the agent choice for (A) each species and (B) each chimpanzee participant, according to mean prosociality rating of the event. The posterior probabilities were obtained from the model with the lowest elpd and highest stacking weight (i.e. participants’ species along the mean rated prosociality of the interaction; see the electronic supplementary material, table S4 and figure S4). The dashed lines correspond to a random choice (0.5). The thick lines represent the grand means and ribbons indicate (from the line) the 30, 60 and 90% credible intervals, respectively. The dots represent (A) the average choice observed across the 16 human adults and the four chimpanzees tested, and (B) the choices of each chimpanzee.

When considering the model including the participants’ species along the mean rated prosociality of the interaction (model with the lowest elpd and the highest stacking weight (= 0.249); see the electronic supplementary material, tables S4 and S5, figure S4), humans did not show a robustly higher probability to select the agent across all prosociality ratings, as none of the marginal differences between species excluded zero from their 90% CI (electronic supplementary material, figure S5). Human participants also did not differ from the chimpanzee participants (marginal effect: mean Δ_Chimpanzee – Human_ = −0.036, 90% CI = [−0.159; 0.114]; with wider priors: mean Δ_Chimpanzee – Human_ = −0.058, 90% CI = [−0.194; 0.080]; and with narrower priors: mean Δ_Chimpanzee – Human_ = 0.016, 90% CI = [−0.099; 0.150]; figure 3), with only 64.97% of the posterior distribution supporting a species difference. Together with the fact (noted before) that the model with species performed decisively less well than the null model without species, this suggests very weak evidence. The absence of an effect was unlikely owing to low sample size and power. This is because a comparison of prior and posterior predictions (electronic supplementary material, figure S2) showed that the model learnt from the data and did not simply return the prior. Therefore, we are relatively confident that there are no species differences in our data.

To assess concerns about the large number of parameters given the sample size, we also conducted a sensitivity analysis by removing all adjustment variables (both fixed and random effects). These simplified models failed to fit the data as well as our main model (model including only the focal parameters (participant species and mean prosociality rating): Δelpd = −197.6, s.e._Δelpd_ = 20.3, stacking weight = 0.06; model including these focal parameters with participants’ and clips’ identities as random effect: Δelpd = −53.3, s.e._Δelpd_ = 11.6, stacking weight = 0.11).

The model with the second-highest weight (stacking weight = 0.187; electronic supplementary material, figure S4), which included the conspecificity of the actors in interaction with the participant species along the prosociality rating of the interaction, did not reveal any robust effect of participants’ species or actors’ conspecificity on the agent choice across all prosociality ratings (electronic supplementary material, figure S6). It also revealed no robust effect of conspecificity (marginal effect: mean Δ_Conspecific - Heterospecific_ = 0.019, 90% CI = [−0.008; 0.046]), species (marginal effect: mean Δ_Chimpanzee – Human_ = −0.045, 90% CI = [−0.172; 0.105]), or their interaction (marginal effect: *humans* mean Δ_Conspecific – Heterospecific_ = 0.020, 90% CI = [−0.012; 0.051]; *chimpanzees* mean Δ_Conspecific – Heterospecific_ = 0.017, 90% CI = [−0.032; 0.064]). Additionally, the actual species of the actor in the videos did not exert a robust influence on the choices (see the electronic supplementary material, S3). Overall, this suggested that while prosociality positively influenced agent choice, species of the participant and whether the actor was a conspecific were not decisive factors.

### 3.2. Other effects

When considering all posterior estimates of the model with the lowest elpd (see the electronic supplementary material, table S5 for all posterior estimates details), the model revealed that, like for the control trials (see the electronic supplementary material, S2 and table S6), the centrality of the agent positively influenced its probability of choice (posterior mean *P*_Agent more centred_ = 0.470, 90% CI = [0.157; 0.785], P(*P*_Agent more centred_ > 0) = 0.99). In addition and contrary to the control trials, the last actor to move and the difference of movement between the agent and the patient positively influenced agent choice (posterior mean *P*_Agent and patient stopped to move with the video_ = 0.438, 90% CI = [0.101; 0.771], P(*P*_Agent and patient stopped to move with the video_ > 0) = 0.98; posterior mean *P*_Agent/Patient move as much_ = 0.599, 90% CI = [0.190; 1.005], P(*P*_Agent/Patient move as much_ > 0) = 0.99; posterior mean *P*_Agent move more_ = 0.629, 90% CI = [0.197; 1.061], P(*P*_Agent move more_ > 0) = 0.99; posterior mean *P*_Agent move a lot more_ = 1.026, 90% CI = [0.410; 1.635], P(*P*_Agent move a lot more_ > 0) = 1), suggesting potential effect.

## 4. Discussion

Video clips with different social interactions, ranging from antisocial (e.g. aggressive) to prosocial (e.g. cooperative), were shown to chimpanzees and humans, two closely related hominids. Both species showed a similar sensitivity to prosociality, exhibiting increased agent preference as prosociality increased. No statistically distinguishable patterns of preference were found between species, nor was there a statistical effect of the actors’ species. We regard this as a relevant finding for a debate that has been ongoing for decades about whether chimpanzee and human psychologies differ in fundamental ways, i.e. whether a contrast between competitively minded chimpanzees and cooperatively minded humans is warranted (e.g. [14]).

Contrary to current thinking, our data did not reveal a meaningful species difference in how subjects perceived social interactions. This finding aligns with previous reports of chimpanzees being sensitive to emotional states of others [29,34,36,37], making it unlikely that they are unable to recognize prosocial behaviours or that they are indifferent to the consequences of prosocial acts, as suggested in various benefit-choice experiments [59–61]. Instead, both species responded to prosocial agents in similar ways, including social interactions with non-conspecific actors. Our findings are more consistent with reports from the wild where prosocial behaviours can be observed (e.g. [43–46]), possibly because individuals depend on one another [67]. They also align with part of the experimental literature that stresses the prosocial nature of chimpanzees. For example, captive chimpanzees occasionally help each other [98], remained highly aroused when unable to do so [99], reciprocate help received from others [54–56], even at material costs [53] and provide resources to others at a personal cost [51].

Equally significant, and in contrast to our predictions and to previous findings [29,30,32], chimpanzees in our study did not exhibit a pronounced inclination towards aggressive, antisocial agents. A recent example from Krupenye & Hare [65] found that bonobos, the other *Pan* species, preferentially chose to receive food from an agent that hindered others compared to a helping agent. This was in contrast to human infants, who, from a very young age, showed high preference for helpers over hinderers [20]. In our study, participants were not required to make direct choices between prosocial or antisocial agents; instead, they could choose between an agent who had acted prosocially or antisocially on a patient and the patient who had undergone this action, a possible key difference. This absence of preference may also be owing to the brevity of the scenes, the moderate level of aggression depicted or the lack of context. For example, aggressive agents may be perceived differently if prior interactions between the actors were presented that explained the aggression. Nevertheless, our findings align with those of Mendes *et al*. [100], who reported that both chimpanzees and humans, from a young age, process prosociality in similar ways and demonstrate principles of fairness judgement [100]. In this way, chimpanzees mirrored human behaviour, which challenges another prevailing argument in the comparative cognition literature, i.e. that an evolutionary change in the human psychology including a basic prosocial motivation, was responsible for human hyper-cooperativity [14].

It is important to note that the choices made by the participants in the present study were construed as indicative of an underlying preference for one actor over the other, suggesting a preference for either prosocial or antisocial behaviours. Recognizing the challenge of isolating a preference for prosociality from a preference for specific physical or psychological features, this study was designed accordingly. The large number of stimuli coupled with their variability aimed to mitigate the influence of individual preferences for particular features, thereby allowing generalizations to be drawn regarding a preference for prosocial (or antisocial) actors.

However, we cannot rule out the possibility that the observed pattern resulted from an avoidance of antisocial agents rather than a preference for prosociality. Testing this assumption with alternative methods, such as eye-tracking, could provide more precise insights into avoidance behaviours. Additionally, the observed preference may have been influenced by a general attraction to motion, as our findings indicate that movement differences and the last actor to move influenced the probability of choice. While these factors may inform decision making, in our opinion, they are unlikely to be the sole drivers, as the resulting pattern would probably remain constant across events, regardless of prosociality.

Overall, our findings align with the current literature on affective perception but contrast with the assumptions drawn from research in which subjects were required to act in cooperative or competitive ways. This suggests that the key difference between humans and chimpanzees may not lie in how they perceive and categorize actions, but rather in the choices the two species make afterwards. This in turn may be determined by their socio-ecological environment and degree of interdependence [67]. In the wild, chimpanzees cooperate to hunt [40–42] and coordinate during territorial defence, as intergroup conflicts can be lethal [40]. Another example comes from the Taï Forest (Ivory Coast) where chimpanzees coexist with leopards, which may have led to the high adoption rates of orphaned immatures, even by unrelated group members [43]. By contrast, captive individuals rarely find themselves in situations where the welfare of others is a concern. They do not depend on others for basic activities, such as finding food or nesting areas, and the loss of an individual has no impact on others’ survival. More generally, this lack of interdependence in captive individuals may prevent the expression of altruistic or prosocial propensities [43] in naturalistic interactions (i.e. non-artificial testing).

Despite the similarities reported in our study, there is no doubt that humans are special in their cooperative tendencies [14]. Humans can act altruistically and cooperate at very large scales, much beyond the dyadic interactions seen in many animals—the main reason for the population expansion and biological success of our species over the last ten thousand years [12,15]. From this perspective, we found it surprising that we did not find a strong preference for prosocial agents over antisocial ones in our human participants, in contrast to previous findings [77]. A possible explanation is that different participant groups completed the touchscreen task and video ratings, with perceptions of prosociality potentially differing between them. However, we consider this explanation unlikely, as both groups were recruited from the same population and video ratings showed excellent interrater reliability, suggesting strong consensus. This consistency makes it improbable that group differences in perception account for the absence of strong prosocial preference in our results.

Another line of explanation is that our choice of antisocial behaviours was mild (to avoid high arousal in subjects) while our choice of prosocial behaviours was simple (to be understandable by all subjects), which reflected everyday situations very well, but may have under-reported eventual species differences at both ends. Here, it would be relevant to compare responses to more extreme events, such as one individual rescuing another from drowning or from the grip of a predator. Whatever such research will reveal, the current hypothesis is that any eventual species differences may not be in terms of how such interactions are perceived, but in how individuals act upon them afterwards. This, we would argue, will depend mostly on the level of interdependence between the members of a social unit, rather than on the species. Additionally, future research may want to test subjects at different facilities, which would further allow studying the effects of group-level differences in interdependence and social tolerance, factors that are likely to influence prosocial behaviour and its perception [46,51,67].

Regarding study limitations, it is obvious that our chimpanzee sample was small and thus not representative of the species as a whole. From the seven chimpanzees that were interested in participating in the study, only four successfully reached the criteria for the testing phase. The human sample was also small and contained a gender imbalance, although this was unlikely to be important [77]. We addressed the sample size limitation by using a Bayesian framework [91] and provided evidence that, despite small sample sizes, our models were able to learn from the data.

To conclude, this study sheds light on the perception of prosociality, which revealed comparable agent preferences in humans and chimpanzees across different degrees of prosociality and different depicted species. Since the chimpanzees’ preference for prosocial agents did not differ in any significant way from humans, we believe that their prosocial psychology may have been underestimated in previous laboratory experiments, which primarily relied on paradigms requiring action-based decisions. Our findings suggest that the gap between humans and their closest living relatives may not stem from differences in action perception but rather from the decisions and behavioural responses that follow.

## Data Availability

Data and code are available at [101]. Supplementary material is available online [102].
